# Seedling mortality in arbuscular mycorrhizal systems

**DOI:** 10.1093/ismejo/wraf285

**Published:** 2025-12-23

**Authors:** Stavros D Veresoglou, John M Halley, Hans Lambers

**Affiliations:** State Key Laboratory of Biocontrol, School of Ecology, Sun Yat-sen University, Shenzhen 518107, China; Department of Biological Applications and Technology, University of Ioannina, Ioannina 45110, Greece; School of Biological Sciences, University of Western Australia, Perth, WA 6009, Australia; Beijing Forestry University, Beijing

**Keywords:** agriculture, Glomeromycota; mycorrhizal responsiveness, crop domestication, seedling mortality, source-sink dynamics

One of the most controversial principles in evolutionary biology concerns how mutualisms persist, despite their susceptibility to cheating. The apparent mechanism that prevents cheating is sanctions. For example, legumes can cut off the oxygen supply to nodules that do not deliver sufficient nitrogen [1], and figs are aborted by the plant host, if fig wasps did not fertilize them [2]. It is comparatively easy to draw conclusions in cases where the endosymbionts belong to a single species, such as those mentioned above, and the host can directly sanction them. However, more complex systems, in which hosts can be conveniently viewed as meta-communities of diverse endosymbionts [3, 4], should experience massive implications as well, which may be exacerbated by common modern practices.

The first plants that colonized the land surface relied on ancestral states of arbuscular mycorrhizas to meet their nutrient demands. Arbuscular mycorrhizas are symbiotic interactions between plant roots and fungi, in which a single host may simultaneously be colonized by multiple species of arbuscular mycorrhizal fungi (AMF), which represent the endosymbionts [4], but, at the same time, hosts of diverse species can be interconnected via common mycelial networks [5]. Plant hosts still benefit from this symbiosis today [6], but there are conditions under which symbiotic costs outweigh benefits [7]. The proportion of terrestrial plants associated with AMF has declined over evolutionary time. The inconsistency with which we observe positive net effects of the symbiosis [8] has given rise to several theories that address how mycorrhizas might benefit plant hosts even if the nutritional effects are, on average, negative. Four complementary possibilities are as follows: (i) plants that exhibit arbuscular mycorrhizal symbioses may support seedling growth (at the cost of resources available to mature individuals) via shared mycelial networks [5], (ii) AMF maintain soil fertility by reducing nutrient losses from ecosystems [9], (iii) hosts receive a wide range of non-nutritional benefits in addition to enhanced nutrient acquisition, and (iv) gains in plant host fitness under adverse settings outweigh the fitness losses under normal environmental conditions [10]. In all four cases, the overall net fitness effects for the plant host are ultimately positive, even if in the short-term costs can outweigh the nutritional benefits. We draw attention to the underexplored possibility that plant mortality triggers changes (i.e. beyond the alterations in nutrient availability) that enhance mycorrhizal benefits for the surviving members of a plant population. Our consideration could have the following two implications: (a) we may be underestimating the nutritional benefits of mycorrhizas to plant growth, because in the contexts we usually assess mycorrhizal benefits, either in controlled experiments or in agricultural settings, we artificially minimize plant mortality; (b) mortality considerations imply that common land-management practices in the Anthropocene (such as pesticide applications and fertilization, reducing plant mortality) favor the spread of less mutualistic glomeromycotan species, making the mutualism less beneficial to the plant hosts.

**Figure 1:**
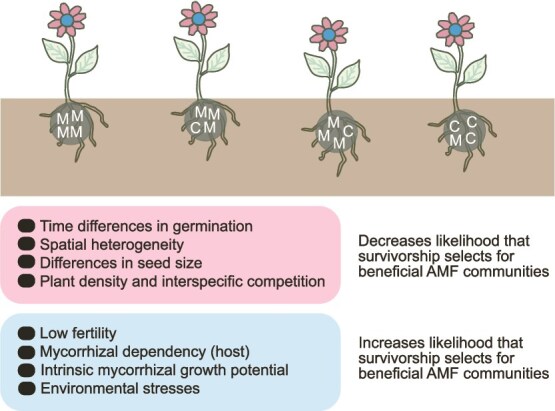
Survival of seedlings can depend on the community structure of endosymbiotic, arbuscular mycorrhizal fungal communities—here we depict them for simplicity as comprising either cheaters (C) or mutualists (M), which we wrote over the root system of the four plant individuals; there can be numerous parameters that determine the degree to which plant mortality may be selecting against cheaters; we present four of the likely mechanisms that reduce the selective potential of mortality (pink balloon), and four (blue balloon) that strengthen it; time differences in germination of conspecific individuals might reduce the impact that mycorrhizal responses have on survival, given that early-germinating plants may have an advantage; the same applies to spatial heterogeneity because plants on nutrient-rich microhabitats are more likely to survive and have seeds with a greater nutrient content; finally, low plant density or extensive interspecific competition with other plants may lower the impact of mycorrhizal responses on host survival; the opposite happens in habitats with a relatively low fertility, or for hosts that depend strongly on mycorrhizas for survival; also, a high intrinsic mycorrhizal growth potential that we observe for some plant species, even if they are not mycorrhizal responsive, opens up opportunities for mycorrhizas to determine survival; finally, in unfavorable environmental settings, because of increased mortality, mycorrhizas are more likely to determine survival.

A contentious issue in mycorrhizal ecology is defining “cheating.” It is possible to describe cheating as the act of consuming resources without giving something in return, which is close to the definition of Johnson *et al*. [7]. Alternatively, cheating can simply describe bad trade rates of nutrients per unit carbon [11]. The exact way through which resources are exchanged in symbiosis [11, 12] has not been fully resolved, and this is the basis of much controversy. From the perspective of theoretical ecology, here we describe “cheaters” as less mutualistic AMF than “mutualists.” This definition encompasses all likely types of cheating and acknowledges that it is beyond the scope of the present perspective to address the underlying mechanisms through which cheating is taking place. We first define the “dead giveaway.” Plants in grassland ecosystems experience mortality rates that exceed 50% of the germinating individuals before reproduction [13]. This figure is even higher in woody ecosystems. Veresoglou and Halley [14] suggested that the death of young plant individuals selects for individuals that have associated with more mutualistic sets of AMF. If the relative abundance of cheaters contributes, even a little, to the mortality of plant hosts, it represents a mechanism through which plant hosts can control the population of cheaters (Fig. 1). Mycorrhizal structures represent a considerable investment for any AMF. Their likely collapse, even when they are part of a larger mycelial network, should compromise growth prospects and possibly subsequent sporulation, but there is a paucity of information on the size of common mycelial networks or the carbon economy of AMF to effectively support this proposition. To mitigate the risk of host mortality, AMF cheaters may have evolved smaller extraradical mycelial networks, minimizing the resource investment lost when a host dies stochastically, which poses an exciting new hypothesis to test. The death of plant hosts that have stochastically recruited cheaters should thus also be the demise for the cheaters themselves that have invested in developing the mycorrhizal structures. We refer to this “gift” from the plants dying to those surviving as the dead giveaway. A necessary condition for early stochastic recruitment of glomeromycotan isolates to determine the fitness of plant hosts is that early arrivals have privileged access to plant photosynthates, for which we use the term “priority effects.” There is compelling evidence that this is the case in arbuscular mycorrhizas [15, 16]. A major regulator of plant host benefits from the mutualism should most likely be the pool of AMF in the surrounding environment which initiate root colonization. Another condition is that plant hosts should be unable to fully differentiate between AMF cheaters and mutualists which is very likely. The degree to which this assumption holds remains unresolved, even though we know that when AMF systems are spatially segregated the host preferentially allocates resources to the most beneficial fungus [11]. Fitter [12] proposed the nutrient-exchange hypothesis, stating that the exchange of carbon in the apoplast is coupled to that for nutrients, and thus the nutrient exchange favors the most beneficial AM fungi. If this is the case, the second condition is also met: it is highly unlikely that the plant host can fully differentiate (i.e. which is a statement that is open to debate: kindly consult the work from Kiers *et al*. [17]) between AMF. Otherwise, at minimum in ecosystems with diverse AMF communities, it would have been virtually impossible for cheaters to persist! Based on our arguments here, the dead giveaway from dying plant individuals to surviving ones is a plausible idea that captures indirect plant-microbe trophic cascades.

**Table 1:** A list of six hypotheses addressing how agriculture and the resulting declining mortality rates across angiosperms might affect the mycorrhizal ecology of the plant hosts (second column) and some ideas to experimentally test them (third column).

Hypothesis	Postulate	Proposed experimental action
Observational (I)—*crop mortality hypothesis*	Crops also experience low mycorrhizal benefits because of the low mortality hosts face in these systems	Compare mycorrhizal responses of crops in soils after adverse environmental conditions leading to mortality rates around ~50% with respective rates under usual conditions.
Observational (II)—*proximity to farms hypothesis*	The net mycorrhizal benefits in natural habitats close to agricultural areas are lower than in comparable areas far away from them	Compare mycorrhizal growth responses of soils with comparable soil properties near farms and far away from farms.
Implications (I)—*extreme agricultural conditions hypothesis*	In extreme environments and across woody habitats (through extensive self-thinning and thus high mortality), the payoffs of the plants in relation to their mycorrhizal associates increase, because mortality of plant hosts also increases	Manipulate mortality rates under such “extreme” conditions and compare mycorrhizal responsiveness at low versus normal mortality rates.
Implications (II)—*mycorrhizal bias of controlled studies hypothesis*	Controlled studies underestimate the benefits from mycorrhiza because they are typically carried out under settings of a very low mortality	Assess mycorrhizal benefits via a rotating cores approach under grassland settings and compare them to the respective figures reported under controlled and agricultural conditions
Evolution (I)—*the mycorrhizal penalties for annuals hypothesis*	Lower mortality rates in annuals might contribute to the low mycorrhizal responsiveness we observe in most annuals.	Under controlled conditions, manipulate mortality rates in perennials versus annuals and assess how these change.
Evolution (II)—*the mycorrhizal domestication hypothesis*	There is a decline in mycorrhizal responsive plant species in the Anthropocene.	Assess palaeoecological records for the relative success of mycorrhizal responsive plant hosts before the introduction of agriculture in relation to existing communities

Why is the above consideration on mortality (i.e. the dead giveaway) important? Agriculture may have a history of only about 10 000 years. However, it is estimated that over 37% of the Earth’s land surface is nowadays used for agricultural purposes. A particularity of agricultural landscapes is that plant mortality is managed to stay exceptionally low by various means, such as pesticides, fertilization but also genetic engineering [18]. This is also the case in controlled experiments, which we often use to measure mycorrhizal benefits to plant hosts. There is compelling evidence that agricultural practices and crop breeding remove the selection for crop lines that benefit from AMF, resulting in crops that no longer benefit from mycorrhizal fungi [19], but there is also an even darker side to crop systems, on which we report right here. If through lowering host mortality we are depriving the plants from a key mechanism to control AMF cheaters, then these should propagate not only in agricultural ecosystems but also, by dispersal into surrounding grasslands and forests. Thus, habitats lying close to croplands could be colonized by a greater proportion of cheaters and receive less positive mycorrhizal benefits than their counterparts far away. Through lowering the mortality rates of the plant hosts, in the Anthropocene, we may be rendering the arbuscular mycorrhizal mutualism less beneficial for plant hosts and interfering with the evolution of terrestrial plants. Moreover, most mycorrhizal benefits have been assayed under controlled conditions or in agricultural settings. Existing meta-analyses synthesize mycorrhizal responses, either in the form of controlled experiments (the vast majority of such studies) or agricultural trials [6]. Given that fungicide additions have many unwanted side-effects, such as non-specific suppression of other soil fungi and being readily mineralized, there are only a few ways of assessing mycorrhizal responsiveness (i.e. biomass gains for the plant host when grown with versus without AMF) in natural habitats. Two effective ways to do so are through rotating cores and plant–soil feedback experiments using the soil in question. Unfortunately, neither of these approaches is widely used in mycorrhizal ecology. We may thereby consistently be underestimating the full potential of mycorrhizas to support plant growth.

How much evidence is there that plant mortality interferes with mycorrhizal benefits? We know that responses of crops to AMF inoculation vary widely [7, 20], and we believe that the indigenous mycorrhizal community may be the cause. It has been proposed that agriculture favors AMF species with aggressive colonizing traits (here defined as AMF with a superior ability to outcompete other AMF for colonization space in the root). This is usually attributed to the high fertility of agricultural landscapes and the use of inorganic fertilizers [6]. How likely is it that this is also driven by the structure of indigenous mycorrhizal communities? Because of a lack of evidence, we will speculate a little here. Neuenkamp *et al*. [21], through a meta-analysis, showed that the benefits from AMF inoculation increase over time which might be because the prevalence of cheaters declines in indigenous AMF pools. Also, Zhang *et al*. [22] observed pronounced differences in the AMF isolates they used for inoculation of populations of *Plantago lanceolata* in self-thinning (i.e. experiencing considerable mortality) studies. However, they noted also that the mycorrhizal responses were greater in the self-thinning treatment compared with the low-density treatment. We therefore think that much of the decline in mycorrhizal responsiveness in agricultural settings is due to the prevalence of cheaters initially present in the local pool of AMF species.

There are several cases in plant and animal ecology where responses are detrimental for the individual but beneficial from an evolutionary perspective such as senescence, sexual reproduction, behavioral altruism, and the overproduction of offspring. We here present through the dead giveaway a likely case study for a microbial system. Most studies addressing anthropogenic activities on microbes focus on drivers of global change and the likelihood that some microbial taxa go extinct [23, 24]. Here, we consider instead the implications of the expansion of agriculture. How might mycorrhizal systems respond to agriculture? (1) An obvious possibility is that the smaller mycorrhizal benefits may trigger a decline in the relative success of mycorrhizal-responsive plant species. Some characteristic species showing a high mycorrhizal responsiveness are legumes, C_4_ grasses, and hosts with “magnolioid” roots [25]. (2) Another possibility is that lower mycorrhizal benefits might trigger lower plant fitness and further investment in early reproduction [26]. The outcome would be shorter generation times compared with that of plant hosts with full mycorrhizal benefits. (3) As a result of declining mycorrhizal benefits, plants might invest further in do-it-yourself strategies (i.e. meet their nutrient demands without mutualists). This would lead to the root systems of many angiosperms becoming more fibrous for greater nutrient scavenging [27]. (4) A final expectation is based on the fact that mycorrhizal benefits vary stochastically more in space than they did in the past (i.e. the idea we develop here). This might open up more opportunities for sympatric isolation and thus evolution. While all four of these points are plausible, we came up with six hypotheses that can readily be tested as to how agriculture and the resulting declining mortality rates across angiosperms might affect the mycorrhizal ecology of the plant hosts (Table 1). We think that addressing those hypotheses represents an important avenue for making advances in mycorrhizal ecology.

We communicate here some ideas on how plant mortality might cascade on the way mycorrhizas function. The ideas we propose can readily be tested and might change how we think of plant and mycorrhizal ecology. We make through our contribution a call for testing these ideas.

## Data Availability

Data sharing not applicable to this article as no datasets were generated or analysed during the current study.
